# Xenotropic Murine Leukemia Virus–related Gammaretrovirus in Respiratory Tract

**DOI:** 10.3201/eid1606.100066

**Published:** 2010-06

**Authors:** Nicole Fischer, Claudia Schulz, Kristin Stieler, Oliver Hohn, Christoph Lange, Christian Drosten, Martin Aepfelbacher

**Affiliations:** University Medical Center Hamburg-Eppendorf, Hamburg, Germany (N. Fischer, C. Schulz, K. Stieler, M. Aepfelbacher); Robert Koch-Institute, Berlin, Germany (O. Hohn); Leibniz-Center for Medicine and Biosciences, Borstel, Germany (C. Lange); University of Bonn Medical Centre, Bonn, Germany (C. Drosten)

**Keywords:** XMRV, respiratory tract infection, immunosuppression, HIV/AIDS and other retroviruses, viruses, expedited, dispatch

## Abstract

Xenotropic murine leukemia virus–related gammaretrovirus (XMRV) has been recently associated with prostate cancer and chronic fatigue syndrome. To identify nucleic acid sequences, we examined respiratory secretions by using PCR. XMRV-specific sequences were detected in 2%–3% of samples from 168 immunocompetent carriers and ≈10% of samples from 161 immunocompromised patients.

Xenotropic murine leukemia virus–related gammaretrovirus (XMRV) was originally discovered in tissue from patients with familial prostate cancer homozygous for a missense mutation in the RNase L gene, R462Q (*1*). Detection of viral nucleic acid in tissue sections of cancerous prostate glands and cloning of the viral integration sites confirmed XMRV as a bona fide human infection with a murine leukemia virus–related retrovirus (*1*). Whether XMRV is actively involved in prostate cancer tumorigenesis or whether it is just a bystander virus (*2*,*3*) remains unclear.

On the basis of its close homology (up to 94% nt identity) to endogenous and exogenous full-length sequences from *Mus musculus* mice (*1*), XMRV most likely originated in mice, although they are probably not the current reservoir of infection (*4*). Recent findings of XMRV sequences in up to 67% of peripheral blood mononuclear cells (PBMCs) of patients with chronic fatigue syndrome and in 3.4% of PBMCs of healthy controls raise the question whether XMRV could be a blood-borne pathogen (*5*). However, the finding of XMRV in PBMCs from patients with chronic fatigue syndrome is controversial because multiple studies in Europe have failed to detect XMRV (*6*–*8*). Similarly, frequency of XMRV in prostate cancer samples ranges from 0 to 23%, depending on geographic restriction of the virus or, more likely, diagnostic techniques used (PCR, quantitative PCR, immunohistochemistry) (*1*–*3*,*9*,*10*). Indirect evidence has suggested sexual transmission (*9*). Questions remain about worldwide distribution, host range, transmission routes, and organ tropism of the virus. To begin to answer some of them, we looked for XMRV in respiratory samples from 267 patients with respiratory tract infection (RTI) and 62 healthy persons.

## The Study

During 2006–2009, the 267 samples were collected from 3 groups of patients (Table). Group 1 comprised patients who had traveled from Asia to Germany; location of their permanent residency was unknown. Groups 2 and 3 and the control group comprised only persons from northern Germany. From group 1, a total of 75 sputum and nasal swab specimens were collected from patients who had unconnected cases of RTI and who had recently traveled by air (*11*). From group 2, a total of 31 bronchoalveolar lavage (BAL) samples were collected from patients with chronic obstructive pulmonary disease (defined by a forced expiratory volume in 1 second/forced vital capacity <70% and forced expiratory volume in 1 second <80% of the predicted value) who had signs of RTI. From group 3, a total of 161 BAL and tracheal secretion samples were collected from patients with severe RTI and immunosuppression as a result of solid organ or bone marrow transplantation. From the control group, throat swabs were collected from 52 healthy persons and BAL samples were collected from 10 healthy volunteers who had no signs of RTI and no known underlying disease.

**Table:** Detection of XMRV in respiratory tract secretions from 329 persons*

Group	Patient median age, y	Underlying disease	Sample	XMRV+
1 (75 patients with RTI)	42	None	Sputum, nasal swab	3/75 (2.3%)
2 (31 patients with RTI)	60	COPD	BAL	1/31 (3.2%)
3 (161 patients with RTI)	32	Immunosuppression after SOT or BMT	BAL, TS	16/161 (9.9%)
Control (62 persons with no RTI)	35	None	BAL, throat swab	2/62 (3.2%)

*XMRV, xenotropic murine leukemia virus; +, positive for XMRV–specific sequences by PCR; RTI, respiratory tract infection; COPD, chronic obstructive pulmonary disease; BAL, bronchoalveolar lavage; SOT, solid organ transplantation; BMT, bone marrow transplantation; TS, tracheal secretion.

All samples were analyzed by culture for pathogenic bacteria and fungi and by PCR for rhinoviruses, adenoviruses, enteroviruses, influenza viruses A and B, parainfluenza viruses 1–3, respiratory syncytial virus, cytomegalovirus, Epstein-Barr virus, and human metapneumovirus. All samples were tested in duplicates obtained by individual RNA extractions. XMRV RNA was reverse transcribed from total RNA, after which nested PCR or real-time PCR were conducted as recently described (*1*,*12*). No serum samples were available from these patients to confirm the results by serologic testing.

For group 1, XMRV-specific sequences were detected with relatively low frequency (2.3%). For group 2, XMRV-specific sequences were amplified in 1 BAL sample, which was also positive for *Staphylococcus aureus* by routine culture methods. For group 3, XMRV-specific sequences were detected at a frequency of 9.9%, which was significantly higher than that for the healthy control group (3.2%) at the 90% confidence level but not at the 95% level (p = 0.078, 1 sample *t*-test). Of 16 group 3 samples, 10 showed no signs of co-infection. The remaining 6 samples showed co-infection with rhinovirus or adenovirus (1 sample each); *S. aureus* (3 samples); or mixed infection with pathogenic fungi, *Candida albicans* and *Asperigillus fumigatus* (1 sample).

All samples that were positive for XMRV by gag-nested PCR, together with a set of those that were negative for XMRV, were retested by real-time PCR. Results showed low XMRV RNA concentrations, 10^3^ –10^4^/mL of specimen.

To confirm the validity of XMRV detection, a subset of 6 specimens (3 XMRV positive and 3 XMRV negative) were tested by using an alternative PCR assay for viral RNA (*3*) and a C-Type RT Activity Kit (Cavidi, Uppsala, Sweden) for type C reverse-transcription activity. XMRV sequences from alternative targets in the gag and env regions were confirmed in 2 of the 3 XMRV-positive samples but in none of the controls. One XMRV-positive BAL specimen showed an 8-fold increase above background of specific type C retroviral reverse-transcriptase activity, suggesting presence of active type C retrovirus within this sample. This assay is substantially less sensitive than reverse transcription–PCR.

All XMRV gag sequences (390-bp fragment) were 98%–99% identical to previously published XMRV sequences from persons with prostate cancer (*1*,*2*). Phylogenetic analysis showed close clustering (Figure).

**Figure:**
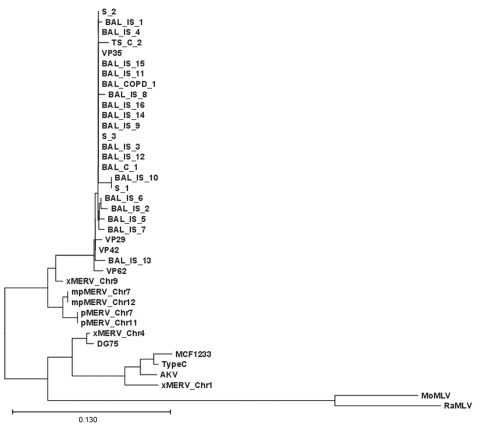
Xenotropic murine leukemia virus–related gammaretrovirus (XMRV) gag sequences derived from respiratory tract secretions. Phylogenetic tree comparing the 390-nt gag fragment of all respiratory samples of this study with recently published XMRV sequences from patients with familial prostate cancer (*1*). The edited sequences were aligned with ClustalX version 1.82 (*13*,*14*) by using default settings. The tree was generated on the basis of positions without gaps only. Sequences are labeled as X, xenotropic; P, polytropic; mP, modified polytropic; S, sputum, IS, immunosuppression; TS, tracheal secretion; and C, control. Scale bar indicates nucleotide substitutions per position.

## Conclusions

XMRV, originally identified in RNase L–deficient patients with familial prostate cancer, has gained interest since recent work showed its protein expression in as many as 23% of prostate cancer cases (*10*) and XMRV-specific sequences were detected in PBMCs of 67% patients with chronic fatigue syndrome (*5*). These results, however, could not be confirmed by others (*6*–*8*). Both studies also detected XMRV protein or sequences in their control cohorts with frequencies of 6% and 4%, respectively.

Among the most pressing information gaps with regard to XMRV is its preferred route of transmission. Detection of XMRV in PBMCs and plasma of patients with chronic fatigue syndrome raises the possibility of blood-borne transmission; sexual transmission has also been hypothesized on the basis of indirect evidence (*5*,*9*). We detected XMRV in respiratory secretions of immunocompetent patients with and without RTI at a frequency of ≈3.2%, which is in good concordance with the recently reported prevalence in the general population of up to 4% (*5*). Frequency of XMRV detection in group 1 patients (2.25%) was comparable to that of human metapneumovirus and rhinovirus within this group and considerably less frequent than that of parainfluenzavirus (15.5%) or influenza A virus (7.6%) detection (*11*).

Our findings indicate that XMRV or virus-infected cells might be carried in and transmitted by the respiratory tract. Attempts to isolate infectious virus from XMRV sequence–positive respiratory samples failed, possibly because of inadequate storage of samples before virus culturing attempts or relatively low copy numbers of the virus within the samples. Thus, whether the respiratory tract serves as a putative transmission route for XMRV cannot be determined at this time. The observed increase in prevalence among immunosuppressed patients with RTI suggests that XMRV might be reactivated in absence of an efficient antiviral defense. Together with earlier observations on increased XMRV replication in RNase L–deficient cells (*1*,*12*), this finding implies that the immune system plays a role in controlling XMRV replication. It remains unknown whether immunosuppression predisposes a patient to secrete infectious XMRV from the respiratory tract or whether presence of virus might be meaningless for epidemiology in a way similar to HIV-1 (*15*). Future studies should address whether the respiratory tract might serve as a source of XMRV infection or whether immunosuppression might cause an increased risk for primary infection.
